# Reductionist Three-Dimensional Tumor Microenvironment Models in Synthetic Hydrogels

**DOI:** 10.3390/cancers14051225

**Published:** 2022-02-26

**Authors:** Rachel R. Katz, Jennifer L. West

**Affiliations:** 1 Department of Biomedical Engineering, Duke University, Durham, NC 27705, USA; rachel.r.katz@duke.edu; 2 Department of Biomedical Engineering, University of Virginia, Charlottesville, VA 22904, USA

**Keywords:** hydrogels, tumor microenvironment, tumor vasculature models, tumor immunity models

## Abstract

**Simple Summary:**

Tumors exist in a complex, three-dimensional environment which helps them to survive, grow, metastasize, and resist drug treatment. Simple, reproducible, in vitro models of this environment are necessary in order to better understand tumor behavior. Naturally derived polymers are great 3D cell culture substrates, but they often lack the tunability and batch-to-batch consistency which can be found in synthetic polymer systems. In this review, we describe the current state of and future directions for tumor microenvironment models in synthetic hydrogels.

**Abstract:**

The tumor microenvironment (TME) plays a determining role in everything from disease progression to drug resistance. As such, in vitro models which can recapitulate the cell–cell and cell–matrix interactions that occur in situ are key to the investigation of tumor behavior and selecting effective therapeutic drugs. While naturally derived matrices can retain the dimensionality of the native TME, they lack tunability and batch-to-batch consistency. As such, many synthetic polymer systems have been employed to create physiologically relevant TME cultures. In this review, we discussed the common semi-synthetic and synthetic polymers used as hydrogel matrices for tumor models. We reviewed studies in synthetic hydrogels which investigated tumor cell interactions with vasculature and immune cells. Finally, we reviewed the utility of these models as chemotherapeutic drug-screening platforms, as well as the future directions of the field.

## 1. Introduction

Solid tumors do not exist as homogenous clusters of cancer cells but rather as complex systems interwoven with vasculature, immune cells, stromal cells, and tumor cell transformed extracellular matrix (ECM) [1,2,3,4]. These systems, referred to as the tumor microenvironment (TME), are critical to the survival, progression, and eventual metastasis of tumors. The TME has also been shown to confer resistance to therapeutics through increased matrix density, immunosuppression, and the induction of drug efflux [3,5].

Despite our increasing understanding of the clinical importance of the TME, in vitro pre-clinical drug screening is primarily conducted on two-dimensional tumor cell monolayers [1,6,7]. This platform removes the paracrine and juxtacrine signaling, matrix interactions, and mechanical properties which are characteristic of tumors. While in vivo mouse models can preserve the three-dimensionality of human TMEs, they do not provide the ability to precisely control individual experimental variables, or the ease of imaging the progression of the TME development. Thus, a reductionist model may be preferable to complex in vivo models when investigating the impact of individual specific properties of the TME on tumor progression. Furthermore, studies of TME-targeting drugs often show poor correlation between outcomes in animal models and what is seen in the clinic, likely due to the inability to accurately recapitulate TME interactions as they occur in humans, especially with regard to immune cells [8]. Across a broad spectrum of pathologies, preclinical studies have suggested that anti-angiogenic therapies—alone or in combination with cytotoxic chemotherapeutics—would be effective, but clinical use has failed [9,10,11]. 

Efforts have been made to circumvent these issues seen using in vivo models via 3D cancer cell cultures in naturally derived biomaterials, such as Matrigel and collagen [12]. These in vitro studies have provided great insight into the difference between tumor cell behavior in 2D and 3D, as well as the roles of secreted factors, 3D paracrine and juxtacrine signaling, and matrix proteins in TME development [7,13]. Natural materials are advantageous in that they are biocompatible and can typically transition from a liquid to a gel of tissue-like stiffness at body temperature under cell-compatible conditions. However, the biochemical and mechanical properties of these materials are intrinsically linked; thus, it is impossible to change their bioactivity without also changing their stiffness or composition, and vice versa [7,12]. In addition, naturally derived materials are subject to a high variability of protein components after isolation from tissue, having as low as 53% batch-to-batch similarity even in growth-factor-reduced formulations [14]. As such, many researchers, especially those who seek to investigate the independent roles of mechanical properties, have shifted to the use of synthetic or semi-synthetic hydrogel platforms. In this review, we will discuss the current state and future directions of reductionist TME modeling using synthetic hydrogels. 

## 2. Commonly Used Materials

A major hallmark of the TME is increased ECM density, leading to a matrix that is both mechanically stiffer and replete with more bioactive sites such as adhesion ligands and enzyme-degradable motifs than the matrix of a comparable healthy tissue [1,4]. Thus, control over the mechanical properties, adhesivity, and degradability of ECM-mimicking materials is important for the design of TME models.

### 2.1. Semi-Synthetic Polymers

One method of overcoming the challenges associated with naturally derived matrices is the synthetic modification of a natural polymer. This is advantageous because it preserves matrix biocompatibility while allowing greater control over composition and mechanical properties. 

#### 2.1.1. Modified Gelatin

Gelatin, which is a completely denatured version of collagen type I, is an attractive backbone cell culture scaffold because of its chemical similarity, increased solubility, and cost effectiveness compared to collagen [15]. Gelatin retains the matrix metalloproteinase (MMP) cleavage sites found on collagen, allowing cell-mediated degradation, thus making it suitable for 3D cell culture. The α_5_β_1_ and α_v_β_3_ integrin binding motif, arginine-glycine-aspartate (RGD), which is sterically obstructed by collagen’s triple-helical structure, is exposed on gelatin [16,17]. Thus, gelatin provides cell binding sites usually seen in fibronectin and the enzymatic biodegradation of collagen in a single matrix component.

In order to increase the stability and tunability of gelatin-based hydrogels, Van Den Bulke et al. pioneered a method to introduce crosslinking sites [18]. Methacryloyl groups are grafted onto the gelatin backbone at primary amine and hydroxyl groups using a direct reaction with methacrylic anhydride. The resulting polymer, GelMA, can be crosslinked into a hydrogel using free radical polymerization [16,18]. A newer, less widely used method to functionalize gelatin with norbornene (NB) groups was developed by the Chin group. Amine groups on gelatin are reacted with carbic anhydride, such that the resultant polymer, GelNB, can be crosslinked into a hydrogel using thiol-ene chemistry [19]. These matrices provide an advantage over more traditional matrices like Matrigel and collagen because they improve batch-to-batch consistency and enable temperature-independent, irreversible, covalent crosslinking [18]. 

The changing of the stiffnesses of GelMA and GelNB matrices can be acheived either by modulating the polymer density or by changing the reaction conditions to create different numbers of methacyloyl groups, and thus different crosslinking densities. A higher polymer amount and higher crosslinking density results in stiffer gels [16]. While these processes increase the experimental control, they do not fully decouple the biochemical and mechanical properties, as an increased polymer density means increased bioactive sites, and an increased crosslink density means the reduced availability of the bioactive sites.

Because of their biocompatibility and tunability, modified gelatin matrices have been successfully used in several in vitro TME models. Examples include the glioblastoma-associated angiogenesis work from the Harley group, which demonstrated that the presence of endothelial cell networks increased the number of glioblastoma cells [20], and ovarian cancer spheroid models from the Loessner group, which evaluated the ability of cells to proliferate and form spheroids in GelMA [21]. The Chin group demonstrated the utility of GelNB as a matrix to study the impact of stiffness and gelatin content on hepatocellular carcinoma cells [22].

#### 2.1.2. Modified Hyaluronic Acid

Hyaluronic acid (HA), a polysaccharide comprised of β (1-4) N-acetyl-glucosamine and β (1-3) linked D-glucuronic acid, is a native component of the extracellular matrix [23,24,25,26]. HA binds cells through CD44, ICAM-1, and RHAMM surface receptors, and is subject to enzymatic degradation via hyaluronidase [24,25,27,28]. As such, it has an inherent biocompatibility that makes it an attractive polymer for 3D cell culture.

In order to create stably crosslinked hydrogels, Smeds et al. developed a method to add methacrylate crosslinking sites to HA polymers [26]. The hydroxyl group on the N-acetyl-glucosamine unit of HA is reacted directly with methacrylic anhydride, grafting methacrylate (MA) groups onto the polymer. HA-MA can then be covalently crosslinked into a hydrogel using free radical polymerization [23,26,29]. A similar process using glycidyl methacrylate with an excess of triethylamine and tetrabutyl ammonium bromide instead of methacrylic anhydride was developed by Leach et al. to yield glycidyl methacrylate HA (GMHA) [23]. A newer HA functionalization method is the reaction of the hydroxyl group of the N-acetyl-glucosamine unit of HA with 5-norbornene-2-carboxylic acid, generating norbornene-HA (NorHA), which can then be crosslinked into a hydrogel via thiol-ene chemistry [30]. The use of modified HA as a tissue engineering scaffold provides the same advantages over traditional matrices as GelMA. Despite HA’s intrinsic cell-adhesive properties, many cell types require integrin activation for long-term survival, such that HA-based matrices often require the incorporation of an integrin-binding peptide like RGD [23,24].

As with modified gelatin matrices, the stiffness of a modified HA hydrogel is typically modulated using the polymer or crosslinking density. Given that HA is biochemically active, the same limitations in the decoupling of mechanical and biochemical cues from HA matrices apply. 

Modified HA matrices have been implemented in many in vitro cancer models. Because of the high concentration of HA in native brain extracellular matrix, it is particularly useful in the study of brain cancer [31]. One example is the Rao group’s work modeling breast cancer metastasis to the brain, which demonstrated that a brain-metastasizing breast cancer cell line preferentially spread and proliferated on hydrogels that mimicked the stiffness of cancerous brain ECM [32]. Many researchers have also chosen to combine HA-MA and GelMA in their scaffolds. Examples include the prostate cancer bone metastasis model developed by the Mano group, which showed that the hydrogel models demonstrated more clinically relevant chemoresistance than spheroids alone [33], and the glioblastoma invasion and angiogenesis models developed by the Harley group, which demonstrated that matrix-bound HA decreased glioblastoma cell invasion and endothelial cell network formation [34,35].

#### 2.1.3. Modified Alginate

Another naturally derived polysaccharide commonly used for 3D cell culture is alginate. It is comprised of (1-4)-linked β-D-mannuronic acid (M units) and α-L-guluronic acid (G units). The G blocks bind cooperatively to multivalent cations, allowing the formation of a hydrogel network between adjacent chains [36,37]. Unfortunately, due to the dissociation of the ions, the mechanical properties of ionically linked alginate are not stable in long-term cultures [36,38]. 

In order to form mechanically stable alginate gels, sites for covalent crosslinking must be introduced. One method, pioneered by the Mooney group, involves adding an N-hydroxysuccinimide (NHS) ester group to the alginate chain [39]. The carboxyl group on the alginate chain is reacted with NHS in the presence of 1-ethyl-3-(3-dimethylaminopropyl), resulting in alginate chains with NHS groups. The hydrogels can then be covalently crosslinked by reacting any dual amine crosslinker with the NHS groups to form amide bonds [39,40]. Another common method of modifying alginate for chemical crosslinking is to add methacrylate groups [41,42]. This process is similar to the modification of HA with methacrylate described in Section 2.1.2. Briefly, the hydroxyl groups on alginate (present on both subunits) are reacted with methacrylic anhydride, generating alginate-methacrylate (AL-MA) which is suitable for free radical polymerization [41]. Like with HA-MA and GelMA, the stiffness of alginate hydrogels is typical modulated using the polymer or crosslinking density.

Unlike gelatin and hyaluronic acid, alginate is derived from algae, and is not naturally found in mammals. As such, it does not have inherent bioactivity that allows it to interact with human cells [36]. This is advantageous in that alginate serves as a “blank slate” for modification with bioactive sites like RGD, and it allows for the independent tuning of bioactive properties and mechanical properties [36]. However, alginate degrades hydrolytically, and is not subject to enzymatic degradation. Because matrix degradation in situ is typically dependent on enzymatic secretion by cells, alginates require further modification to mimic this feature of the native environment [43]. 

Modified alginate matrixes have been used extensively for tissue engineering and drug delivery [44], but are less commonly used for tumor models than GelMA and HA-MA, perhaps due to the importance of enzyme-mediated degradation in tumor progression [45]. Examples of modified alginates used as tumor models include the work from Fischbach et al. which examined the impact of the presence of RGD and 2D vs. 3D culture on oral squamous cell carcinoma growth factor secretion [46], and Grigore et al.’s study on the impact of the addition of RGD and gelatin to covalently crosslinked hydrogels on the behavior of osteosarcoma cells [47]. Both studies found that RGD upregulated growth factor secretion, and Grigore et al. showed that the incorporation of gelatin further upregulated signaling. These studies demonstrate the suitability of modified alginates as platforms to investigate tumor cell–ECM interactions.

### 2.2. Synthetic Polymers

Another approach to overcome the limitations associated with naturally derived matrices is to generate a matrix from a modified bioinert synthetic polymer. Poly(2-hydroxyethyl methacrylate) (PHEMA) and poly(vinyl alcohol) (PVA) are hydrophilic, bioinert polymers which have been used for this application. However, poly (ethylene glycol) (PEG) is considered to be the gold standard because of its superior resistance to protein adsorption (due to its hydrophilicity, chain mobility, and lack of hydrogen bond donating groups), ease of modification, and ability to mimic tissue-level stiffness regimes [48]. Because adhesion and degradation sites must be added to the PEG, these properties can be controlled independently from one another, as well as from the matrix mechanical properties.

#### 2.2.1. Multi-Arm PEG

PEG alone cannot form a hydrogel; it must be modified in order to be able to form a network. One common method of doing so is the use of multi-arm PEG molecules, in which several chains of PEG are covalently linked at one core site, creating a “star”-shaped polymer. These PEG molecules are created by grafting PEG methyl ether methacrylate (PEGMA) chains onto a tripentaerythritol core, with the reaction occurring at the methacrylate site [49]. The free ends of the PEG arms typically end with norbornene groups (PEG-NB) or vinyl sulfone groups (PEG-VS), such that these multi-arm molecules can be connected with crosslinkers via click chemistry [50,51]. Because PEG itself does not contain adhesion or degradation sites, these crosslinkers provide an opportunity to add these modifications. Often, a matrix metalloproteinase-sensitive peptide flanked with cysteine groups is used as the crosslinker, creating a hydrogel PEG network than can be degraded in the presence of cells. To some of the arms, RGD motifs are added, such that there are dangling adhesion groups for cells [51,52]. Thus, cells, multi-arm PEG molecules, and crosslinker can be mixed and polymerized, generating a cell-laden hydrogel.

The mechanical properties of multi-arm PEG gels can be tuned by changing the length of the PEG arms or the density of the PEG molecules, as well as the crosslinker concentration. Additionally, the amount of RGD and/or other adhesion molecules in the gel can be titrated independently of the gel’s density and stiffness. This system has been used to model stromal cell interactions with melanoma cells by the Anseth group, demonstrating that the incorporation of fibroblasts decreased the proliferation and cluster size but increased the invasion of the melanoma cells [53], and to closely mimic the in situ, stiffness, and stromal cell cues on pancreatic ductal adenocarcinoma organoids by the Griffith and Jorgenson groups [54].

#### 2.2.2. PEG-Diacrylate (PEGDA)

Another modification method to make PEG suitable for hydrogel networking is to add acrylate groups on either end. PEG is reacted with acryloyl chloride in a 1:2 molar ratio, creating PEGDA. These groups form micellar crosslinking sites in an aqueous solution, and can be attached covalently via free radical polymerization [55]. In these systems, degradation sites must be inserted in the backbone of the polymer itself. MMP-sensitive peptides are typically conjugated to PEG using an NHS ester leaving group, such as SVA. A 2:1 molar ratio of an acrylate-PEG-SVA to a peptide with a side chain near the C-terminus containing a free amine group yields an acrylate-PEG-peptide-PEG-acrylate chain. These chains can then be crosslinked to form hydrogels which break apart in the presence of MMP-secreting cells. Similarly, RGD or other cell-adhesive peptides can be conjugated to an acrylate-PEG-SVA chain, providing an opportunity to immobilize cell adhesion sites to the hydrogel [56,57].

In this system, the mechanical properties can be modified by changing the backbone density, or by introducing a competing monomer which de-densifies crosslinking sites without significantly changing the mesh size, which would reduce the ability for nutrients to diffuse through the matrix [58,59]. These modifications remain independent of the adhesion site density or the density of any other dangling chain bioactive group. Acrylate-modified PEG hydrogels have been used to model tumor progression in lung adenocarcinoma, which showed that the matrix stiffness and adhesivity influence the EMT and spheroid morphogenesis [12,60,61], and to model dormancy in triple negative breast cancer by the Slater group, which showed that the breast cancer cells could be directed toward growth or dormancy states by the manipulation of the matrix adhesivity and degradability [62,63].

**Figure 1:**
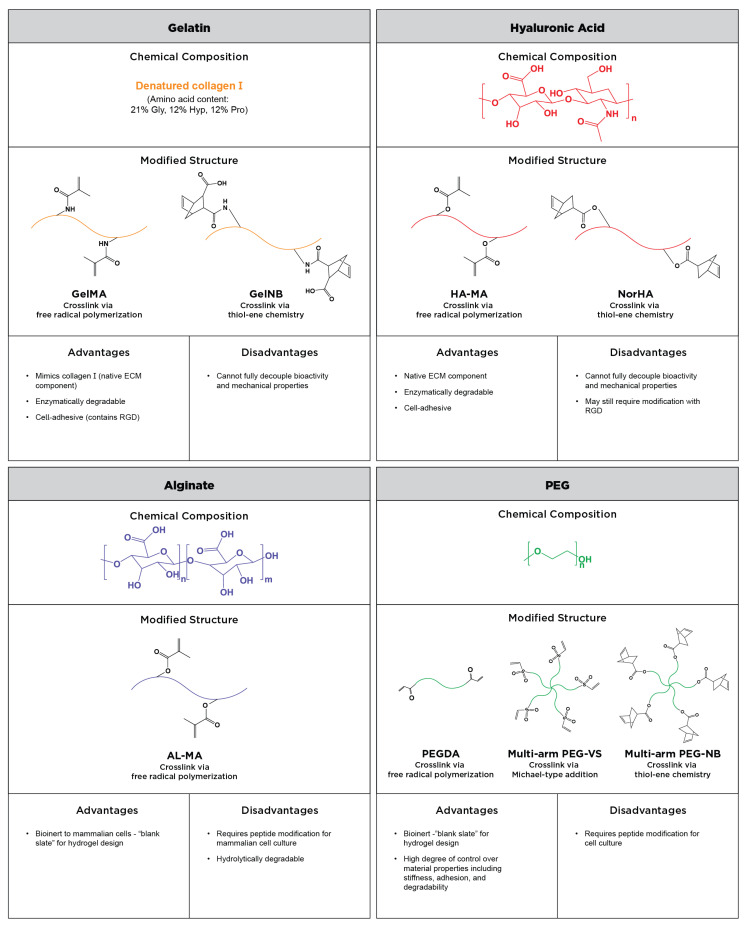
Summary of the commonly used semi-synthetic and synthetic polymers for reductionist tumor microenvironment models. The chemical composition of gelatin, hyaluronic acid, alginate, and PEG are presented, as well as modified structures with sites for crosslinking. The advantages and disadvantages of each material as an ECM-mimetic matrix are discussed.

Most of the aforementioned systems can be used in combination with one another. Some researchers, like Jiang et al., in an osteosarcoma model and the Harley group in a glioblastoma model, have opted to mix GelMA with PEG to use the advantage of the inherent bioactivity of gelatin while also gaining slightly more independent control of the matrix stiffness [64,65,66]. Naturally derived ECM materials such as collagen and Matrigel have also been blended with synthetic or semi-synthetic materials to create hybrid hydrogels [67,68,69]. The composition, advantages and disadvantages, and crosslinking methods of commonly used semi-synthetic and synthetic polymers are depicted in Figure 1.

#### 2.2.3. Emerging Material: Self-Assembling Peptides

While they are not yet widely used in tissue engineering, self-assembling peptide nanofibers are an emerging synthetic biomaterial platform for mammalian cell culture [70,71]. They are composed of lab-synthesized peptides with amino acid sequences designed to assemble into β-sheets and/or α-helices. At high concentrations, these peptides form fibrous hydrogel networks, which can be used to mimic the ECM [71,72,73,74]. The mechanical and chemical properties of these hydrogels are modified by changing the amino acid sequence. Because they are peptide-based, adding in bioactive sites such as RGD is relatively facile; the peptide of interest is synthesized on one end of the self-assembling nanofiber sequence, and is thus incorporated directly into the hydrogel during self-assembly. Due to the ability to form supramolecular structures based on sequence, they don’t require chemical modification in order to crosslink [70,72,73,74]. Recent work from the Hernandez, Miller, and Saiani groups has shown the feasibility of the use of these hydrogels to study the impact of gel stiffness in a pancreatic cancer model, and to study chemoresistance in a breast carcinoma model [73,74]. 

### 2.3. Crosslinking Polymers into Hydrogels

In order to convert a hydrophilic polymer into a hydrogel, the material must be crosslinked into a continuous mesh network. While naturally derived polymers like collagen and Matrigel can form a network by physical entanglement at body temperature, when interactions between the chains are highly favorable [75], synthetic and semi-synthetic hydrogels typically rely on secondary interactions and covalent crosslinking mechanisms [76]. One of the most common methods is free radical polymerization, in which a chemical-, thermal-, or photo-initiated free radical attacks accessible double bonds, breaking them and covalently linking functional groups together [48,56,76]. This method is employed to crosslink GelMA, HA-MA, AL-MA, and PEGDA, as all three of these materials contain acrylate groups, the double bonds of which can be attacked by free radicals. For tissue engineering applications, the photo-initiated generation of free radicals is the most attractive, because it is efficient, and because cells are able to tolerate brief exposure to UV and visible light without losing viability [56]. 

Other common cytocompatible methods for covalent hydrogel crosslinking fall under the umbrella of click chemistry: fast, high-yield, and highly selective chemical reactions which link complementary groups [77]. One such method is Michael-type addition, in which enolate nucleophiles and activated electrophilic olefins react with no initiation required. Like free radical polymerization, this method is highly efficient, but it does have the disadvantage of side-reactions with competing nucleophiles, which are present in the context of live cells and other biological compounds [48,78,79]. Michael-type addition chemistry can be used to crosslink multi-arm PEG-VS [78]. Another commonly used click chemistry method is the radical-mediated reaction between thiol and alkene groups, which is known as ‘thiol-ene’ coupling [50,80]. Thiol-ene chemistry is used to crosslink multi-arm PEG-NB, GelNB, and NorHA [19,30,53,81].

The Burdick group has pioneered a crosslinking method called ‘guest-host’ assembly, which relies upon hydrophobic interactions of adamantane (guest) and β-cyclodextrin (host). Because the mechanism exploits hydrophobic interactions, these hydrogels self-assemble in water but easily disassemble under shear stress [82,83]. This method has primarily been used with adamantane- and β-cyclodextrin-modified HA, but it has recently been employed by the Phelps group to anneal PEG microgels [84].

Because all of these crosslinking mechanisms are cytocompatible, they can be used to encapsulate tumor cells or spheroids in hydrogels. In order to encapsulate single cells, a cell pellet is homogenously mixed with the polymer precursor solution prior to the crosslinking. In order to encapsulate spheroids, the polymer precursor is either pipetted over a pre-formed spheroid, or a pre-formed spheroid is placed into the precursor solution prior to the crosslinking. Once the tumor cell-laden gels are formed, they can be maintained in standard cell culture conditions.

## 3. Modeling Different Aspects of the TME in Synthetic Hydrogels

While traditional tools like conditioned media and transwell co-culture studies have greatly advanced the understanding of cell–cell signaling in cancer, 3D TME models allow the in-depth investigation of multiple avenues at once in a physiologically relevant system. 

### 3.1. Cell–Matrix Interactions

One of the hallmark characteristics of the tumor microenvironment is a transformed ECM, which is denser, stiffer, and contains more bioactive sites than healthy tissue [2,4]. As described in Section 2.2, the mechanical stiffness and presentation of cell binding motifs in PEG hydrogels can be independently modified. As such, these hydrogels are suitable platforms for the examination of the tumor cell response to biochemical and mechanical cues because they allow the separate and interactive investigation of these matrix properties. The West group studied epithelial morphogenesis in lung adenocarcinoma cells grown in PEGDA-based hydrogels. They found that increasing the stiffness of the gels via the PEG backbone density independent of the adhesion ligand concentration resulted in more polarized, homogenous, lumenized spheroids with fewer cells proliferating. They found the same results when increasing the adhesion ligand concentration using PEG-RGDS concentration [61]. The Slater group used a similar PEGDA platform to study the impact of hydrogel degradability, modulated by the incorporation of a small co-monomer, and adhesion ligand concentration, modulated by PEG-RGDS concentration, on tumor dormancy in triple-negative breast cancer cells. They found that, in high degradability/high adhesivity conditions, cells were in a high growth state characterized by high proliferation, high metabolic activity, and low apoptosis. In moderate degradability and adhesivity conditions, the cells move to a moderate growth state. However, cells can be pushed toward dormancy in low degradability conditions, surviving in single-cell restricted dormancy in the absence of adhesion ligands, or balanced dormancy in the presence of adhesion ligands in lower degradability conditions [63]. 

The tunability of PEG-based matrices make them ideal candidates for rational matrix design. In a collaboration between the Griffith and Jorgenson groups, PEG-VS was employed to create a physiologically relevant synthetic matrix for pancreatic ductal adenocarcinoma organoids. They assessed the ECM of neoplastic pancreatic tissue and identified suitable binding motifs, which they incorporated into their hydrogels. The organoids grew just as efficiently in this reductionist model as they did in Matrigel. They then assessed the stiffness of neoplastic pancreatic tissue compared to healthy tissue, and altered the PEG-VS density to achieve matching stiffnesses. They found that, while keeping the ideal binding motifs the same, they saw an increased growth rate of the organoids in stiffened gels (8.2 kPa–20.5 kPa) compared to the softer gels (1.4 kPa) [54]. 

These examples illustrate that synthetic PEG hydrogels are useful for the examination of cell–matrix interactions. Because they are broadly cytocompatible, other cell types can be incorporated in these hydrogels alongside the tumor cells. Thus, cell–matrix and cell–cell interactions can be investigated in concert.

### 3.2. Tumor Vasculature

#### 3.2.1. Current Models

The development of vasculature in the TME is key for tumor growth and survival. The diffusion of critical nutrients limits cell survival to a distance of 200 µm from the nearest blood vessel. When the tumor cell mass grows past this limit, hypoxic conditions trigger cells to release growth factors, such as vascular endothelial growth factor (VEGF) and hypoxia-induced factor (HIF) [12,85]. These factors work in concert to trigger endothelial cells to sprout vessels and recruit stromal cells as vascular support. This shift in expression is known as the angiogenic switch [12], and has been shown to contribute to both chemo- and radioresistance [86,87]. The development of tumor vasculature not only delivers nutrients to the tumor to facilitate growth but also provides routes for tumor cells to escape the primary tumor environment, circulate, and eventually colonize a distal tissue [1,12,60,88]. 

**Figure 2:**
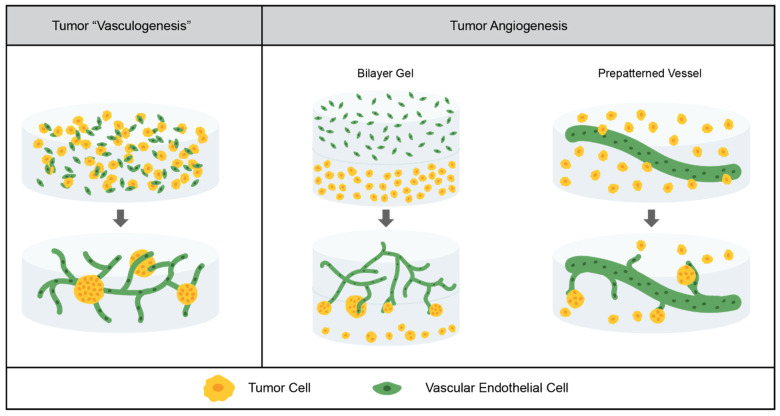
Tumor vasculature models in synthetic hydrogels. Current models have demonstrated tumor “vasculogenesis”, in which already differentiated vascular endothelial cells form networks in the presence of tumor cells, and angiogenesis, in which tumor cells induce sprouting from existing vessels. These existing vessels can be created using a bilayer gel system or a prepatterned vessel.

There are two ways in which blood vessels are formed in situ: vasculogenesis and angiogenesis, as depicted in Figure 2. Vasculogenesis is the de novo formation of vasculature, beginning with the differentiation of precursor cells to endothelial cells, whereas angiogenesis is the formation of vasculature by sprouting from existing blood vessels [88,89]. Tumor vasculature develops through angiogenesis. When the cluster of tumor cells becomes too large for the diffusion of nutrients, the hypoxic environment triggers the angiogenic switch. Tumor cells secrete pro-angiogenic signals—such as VEGF, platelet-derived growth factor (PDGF), and fibroblast growth factor—which induce sprouting from nearby blood vessels [88,90]. 

While angiogenesis is the process that occurs in the TME in situ, synthetic hydrogel models which employ the vasculogenic-like formation of tumor vasculature have helped to advance the field. The Werner group constructed tri-cultures of cancer cells (triple-negative breast cancer or prostate cancer), human umbilical vein endothelial cells (HUVEC), and fibroblasts (which help support and stabilize vasculature) in a multi-arm PEG hydrogel system. Because the vessels in this system form from endothelial cells rather than sprouting from existing vessels, this system does not model angiogenesis. However, because the endothelial cells are already differentiated, it is not quite a vasculogenesis model; rather, it is a mid-point between the two. They found that tumor cells encapsulated in a single suspension grew preferentially close to the vessels, and that pre-grown spheroids induced vasculature formation in contact with the spheroids. In comparison to Matrigel, they saw more ordered spheroid and vasculature formation which was consistent with in vivo models [91].

In order to more directly model tumor angiogenesis, a bilayer model in a PEGDA-based system, in which the top layer contained HUVEC and pericytes to support and stabilize the vasculature, and the bottom layer contained lung adenocarcinoma cells, has been used, as depicted in Figure 2. The vasculature was able to form in the top layer, as had been previously demonstrated [57], and after a tubule network was formed, sprouting toward the bottom layer of tumor-cell laden gels occurred. The cluster area was unchanged, but it was found that the cluster circularity was much lower at the interface of the vascular and tumor gel layers than it was deeper into the vascular gel [12]. In a second study in this bilayer model, the West group evaluated three lung adenocarcinoma cell lines: one highly metastatic, one moderately metastatic, and one nonmetastatic. They found that all of the tumor cell lines induced sprouting, but the highly metastatic cell line induced more sprouting, secreted more pro-angiogenic factors, and showed more dramatic morphogenic change at the interface of the vascular and tumor gel layers [60].

Another method for the modeling of angiogenesis is to coat pre-formed channels with endothelial cells, such that tubules can sprout from these channels into the surrounding gel. In the context of tumor angiogenesis, this method was used by the Yang group. Hydrolytically degradable alginate microfibers were embedded in a multi-arm PEG-NB hydrogel system, providing channels for endothelialized tubes. Glioblastoma cells were embedded in the surrounding gel, such that the interaction between the cells and the hydrogels could be investigated. No support cells were included; at the time scale tested, sprouting was not observed. However, they found that the presence of endothelialized tubes increased the tumor cell proliferation, and that the presence of tumor cells decreased endothelial cell–cell junctions, both of which are phenomena observed in vivo. It was noted that the tubes produced were much larger than the vessels at tumor sites in situ, but this study served as a proof of concept for a platform that could later be used for a more in-depth study of tumor angiogenesis at a relevant size scale and including support cells [92].

#### 3.2.2. Limitations of the Field

There are many great tumor angiogenesis models in microfluidic devices that rely on naturally derived polymers, such as those constructed by the Kamm group [93,94,95,96]. These models are particularly useful in the study of tumor cell intravasation, the process by which cells escape the primary tumor site by migrating into the vasculature, and extravasation, the process by which tumor cells colonize distal sites by migrating out of the vasculature. In order to model intravasation, the Kamm group used a dual-channel microfluidic device embedded with collagen. In one channel, endothelial cells were seeded with tumor cells seeded in a parallel channel across the collagen matrix. The tumor cells were able to migrate across the gel and intravasate into the endothelial channel [93]. In order to model extravasation, endothelial cells were embedded in the collagen matrix, and were allowed to form perfused tubules between the two microfluidic channels. Tumor cells were flowed through one channel, and they were able to observe extravasation through the tubules into the collagen matrix in response to tumor-like flow conditions and, in the case of breast cancer cells, the presence of bone cells in the matrix (bone is a common metastatic site of breast cancer) [94,96].

There are also several examples of microfluidic models of vasculature in synthetic hydrogels. In a collaboration between the Schwartz and Murphy groups, induced pluripotent stem cell-derived endothelial cells (iPSC-EC) were embedded in a multi-arm PEG-NB hydrogel within a microfluidic device. They found that the cells could form stable, lumenized tubule networks without support cells, which is not possible in static culture [97]. Similarly, the West group demonstrated the ability to form stable, perfused vascular networks from HUVECs and 10T1/2 mesenchymal progenitor cells (to act as vascular support cells) in a PEGDA-based system within a microfluidic device [98]. 

**Figure 3:**
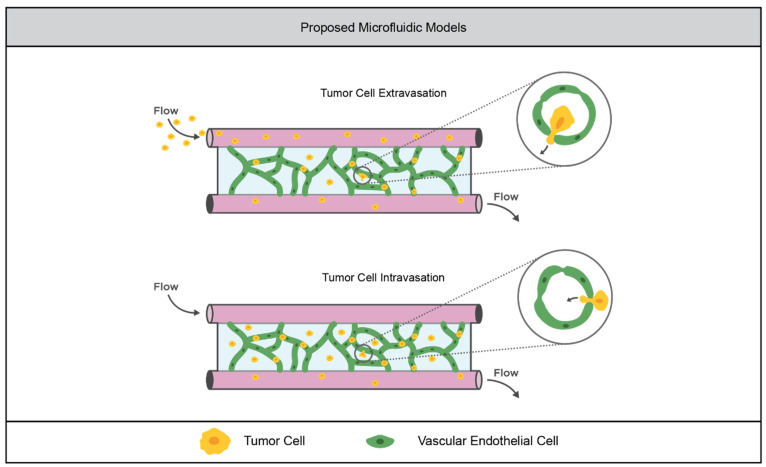
Proposed opportunity for advancement in synthetic hydrogel models of tumor vasculature. The vascular networks formed in synthetic hydrogels within microfluidic devices could enable the investigation of intravasation and extravasation. Tumor cells could be flowed through the vessels, allowing the live imaging of cells extravasating into the matrix. Alternatively, tumor cells could be incorporated into the matrix, allowing the live imaging of cells intravasating into the vascular network.

Despite these advancements, there is a notable absence of microfluidic models for the study of tumor cell intravasation and extravasation in synthetic hydrogels, as depicted in Figure 3. Some of the studies in synthetic hydrogels previously mentioned have demonstrated that tumor morphogenesis is different in synthetic hydrogels compared to naturally derived hydrogels, and the provision of an escape route for eventual metastasis is an important role of tumor vasculature. Thus, it is important for future work in the field of tumor vasculature models in synthetic hydrogels to move toward platforms, such as the microfluidic systems described here, that can recapitulate this behavior.

### 3.3. Tumor Immunity

#### 3.3.1. Current Models

**Figure 4:**
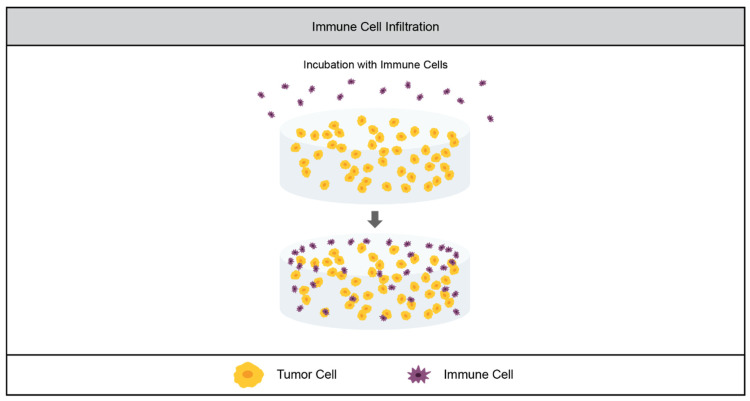
Tumor-associated immune cell models in synthetic hydrogels. The current models of tumor cell interaction with immune cells involve incubating a tumor cell-laden hydrogel with immune cells in order to study immune cell infiltration.

Another aspect of the TME that is key to tumor angiogenesis, progression, and metastasis is the presence of immune cells. Tumor-secreted growth factors recruit immune cells like neutrophils, natural killer cells, monocytes and stromal-resident macrophages to the tumor site [5,90,99,100,101]. While these cells initially surveil the tumor, they are eventually reprogrammed by tumor cells to prevent tumor monitoring, and even to promote survival and metastasis by upregulating pro-growth and pro-angiogenic factors [5,90,100,102]. High densities of tumor-associated macrophages (TAMs) have been shown in clinical studies to be correlated with tumor angiogenesis and poor prognosis [103], as is the case of tumor-associated neutrophils (TANs) [100]. While the field of tumor immunology is rapidly expanding, the study of it in the context of 3D in vitro culture is relatively new. As such, there are fewer examples of synthetic hydrogels being used to study tumor–immune cell interactions than there are of their use to study tumor vasculature [104]. Key immune cells that have been incorporated in TME include NK cells, which initially inhibit tumor growth but are ‘turned off’ by late-stage tumors, and macrophages, which in the M1 phenotype inhibit tumor growth, but promote it when repolarized to the M2 phenotype. The concept of synthetic hydrogels for these studies is shown in Figure 4.

The Sharma group used a PEGDA-based hydrogel system to investigate NK cell invasion in a lung adenocarcinoma model. They evaluated two different cell lines: one highly metastatic and one nonmetastatic. They cultured these cells within the hydrogels for 1 day (early stage) or 7 days (late stage), and then incubated the tumor cell-laden gels with NK cells. The NK cells infiltrated further into the gels with the nonmetastatic cell line than the gels with the metastatic cells, and further into the early-stage gels than the late-stage gels. Furthermore, the metastatic cells and late-stage gels reduced the production of immunomodulatory signals by NK cells. These results recapitulate phenomena observed in vivo, as NK cells are more effective at surveilling early stage, less-aggressive tumors [105].

The effect of M1-polarized macrophages on tumor cells’ growth was studied in a PEGDA-based system in a collaboration between the Kao and Man groups. Macrophages were pre-polarized to the M1 (tumor surveilling) phenotype, and were encapsulated within the hydrogel in a transwell insert. The insert was then placed in a well with a monolayer of either hepatocellular carcinoma cells or a healthy hepatocyte line. They found that the M1-polarized macrophages had an adverse effect on the growth of the tumor cells but not on the transformed cells. While this study served as a 3D model for tumor-surveilling macrophages, it was not a 3D TME model, as the tumor cells were cultured in 2D on tissue culture polystyrene [106]. 

#### 3.3.2. Limitations of the Field

As in the case of tumor vasculature models, there are some models of the immune logical component of the TME that rely on naturally derived models. Examples include work from the Kamm group in which macrophages incorporated in their dual-channel microfluidic platform (Section 3.2.2) enhanced tumor cell intravasation into the endothelial channel [93]. In another model, the group encapsulated macrophages in collagen in their microfluidic model and subjected them to the interstitial flow rates seen in the TME. They found that these flow conditions polarized macrophages toward the tumor-promoting M2 phenotype. When these flow-polarized macrophages were cultured with triple-negative breast cancer cells, they enhanced migration [107]. The Chandrasekaran group constructed a single-channel microfluidic model in which neutrophils were flowed through a porous channel, such that they could migrate into a collagen gel with embedded ovarian tumor spheroids. They found that the tumor spheroids induced neutrophil migration, and vice versa. They also demonstrated that the tumor cells induced the production of neutrophil extracellular traps (NETosis), which had a pro-migratory effect on the tumor cells [108].

There are also synthetic hydrogel models of immune cell behavior without the presence of tumor cells. For example, in a PEGDA-based hydrogel system with macrophages and endothelial cells, M1 macrophages inhibited vessel formation while M2 macrophages promoted it. These macrophages can act as support cells to stabilize the vasculature, or as bridging cells to connect sprouts [109]. Other studies have demonstrated the ability of neutrophils to adhere to and migrate along PEGDA hydrogels modified both with PEG-RGDS and a Mac-1 binding peptide, demonstrating that synthetic hydrogels are suitable for neutrophil culture [110,111]. 

**Figure 5:**
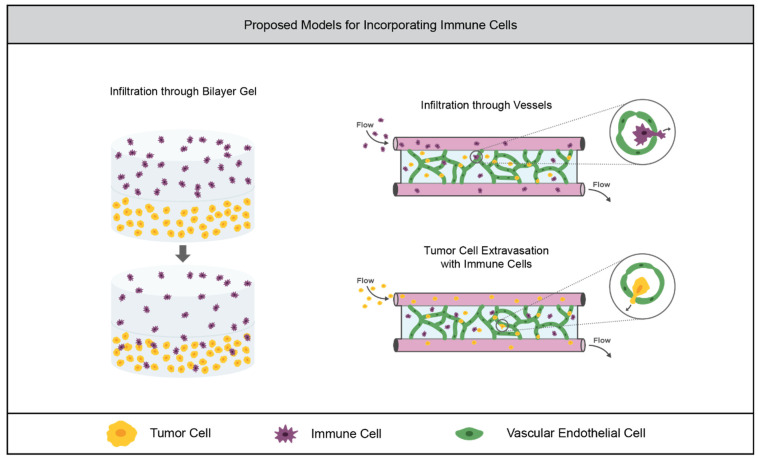
Opportunities for advancement in synthetic hydrogel models of tumor-associated immune cells. Instead of incubating tumor cell-laden hydrogels with immune cells, a bilayer model could be used to mimic immune cell infiltration from the surrounding tissue. Vascular networks formed in synthetic hydrogels within microfluidic devices could enable the investigation of the infiltration of immune cells through the vasculature. Furthermore, immune cells could be embedded in the hydrogel in order to study their effect on tumor cell extravasation or intravasation.

Tumor-associated immune cells have been shown to be compatible with synthetic hydrogel cultures, and TME models in naturally-derived matrices have successfully recapitulated tumor–immune cell interactions. Therefore, there is an opportunity for the further incorporation of immune cells in TME models within synthetic hydrogels. Similar work to the NK cell study by the Sharma group could be performed with neutrophils, macrophages, or other immune cells of interest. This work could also be carried out in a bilayer gel similar to the angiogenesis model (Section 2.2.1), in which immune and tumor cell infiltration across the gel interface could be examined, as depicted in Figure 5. Immune cells could also be incorporated into a microfluidic TME model which uses a synthetic hydrogel system, as depicted in Figure 5.

**Figure 6:**
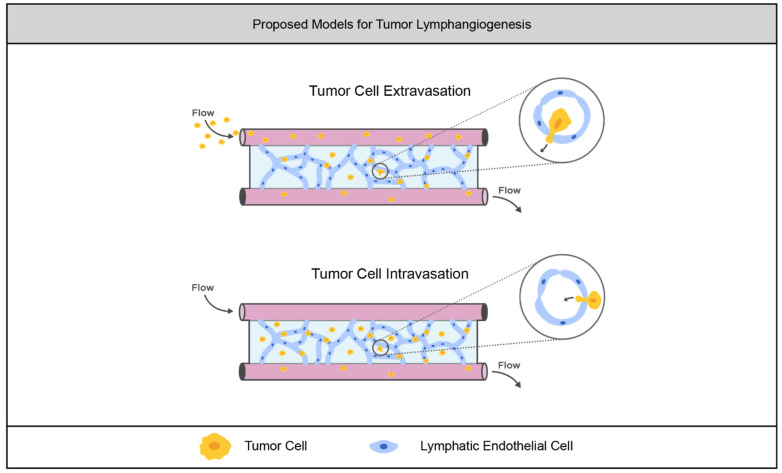
Opportunities to advance models of tumor lymphatic vasculature. Lymphatic vascular networks formed in synthetic hydrogels within microfluidic devices could enable the investigation of tumor cell extravasation from and intravasation to the lymphatic system.

A key aspect of tumor immunology that is relatively unexplored in synthetic hydrogel TME models is the lymphatic system. As with blood vessels, lymphatic vasculature plays important roles in tumor progression, providing routes for cells to escape the primary tumor [103]. Tumor cells also induce lymphangiogenesis, the process by which new lymphatic vessels sprout from existing vessels [112]. Tumor cells use the lymphatic system to suppress tumor surveillance by the immune system [112,113]. The Hagendoorn group developed an immortalized lymphatic endothelial cell (LEC) line by transfecting primary LECs with telomerase. They were able to form stable, perfused lymphatic tubules by embedding the LECs in a collagen matrix within a microfluidic device. When Matrigel containing colorectal cancer organoids was embedded alongside the LEC-laden gel within the microfluidic device, the organoids were shown to increase in size, closely associate with the interface, and induce sprouting from the lymphatic tubules [114]. Recently, the Dixon group demonstrated the feasibility of culturing lymphatic vessels in a synthetic hydrogel system. They embedded sections of lymphatic vessels in a multi-arm PEG hydrogel system, and investigated their sprouting into the gel. They found that the sprouting increased in denser PEG gels and gels with higher adhesion ligand concentrations [115]. These two different models imply the feasibility of the eventual use of synthetic hydrogels to model tumor lymphangiogenesis as illustrated in Figure 6.

## 4. Chemotherapeutic Drug Screening

In addition to providing a useful platform for the investigation of tumor behavior, TME models in synthetic hydrogels can be used to screen chemotherapeutic drugs. There is increasing evidence that there is decreased chemotherapeutic efficacy when they are screened on 3D models compared to 2D monoculture, indicating that the 3D platforms more closely resemble tumor behavior in situ [116,117].

The Werner group screened both chemotherapeutic drugs and anti-angiogenic agents in their triculture model (discussed in Section 3.2.1). They found that chemotherapeutic drugs were less effective at reducing the metabolic activity of cells in the triculture models compared to the 2D cultures, and more metabolic activity was recovered two days post-treatment. They also screened anti-angiogenic drugs on the 3D tricultures and compared them to 3D monocultures. All of the drugs caused the breakdown of the vascular network, but only ataxinib effectively reduced the overall cell metabolic activity in the tricultures. The anti-angiogenic therapies were not effective at reducing metabolic activity in the tumor monocultures, demonstrating the utility of the triculture model as a drug-screening platform [91].

In the NK cell infiltration model from the Sharma group (discussed in Section 3.3.1), the researchers identified that TGF-β was upregulated in the more advanced tumor models where NK cell infiltration was reduced. When they treated the models with a TGF-β inhibitor, they saw that the N cell infiltration in the more metastatic and late-stage models increased to the level of infiltration seen in the early stage, less-metastatic models. Thus, they demonstrated TGF-β as a potential target to reactivate the tumor-inhibiting behavior of NK cells. They also demonstrated the potential for their system to be used as a platform for the identification of therapeutic targets and screening inhibitors [105]. 

As with investigatory models, there is great opportunity for the advancement of synthetic hydrogel TME drug screening platforms using microfluidics. Microfluidic devices with tumor cells embedded in collagen gels have been employed by the Neuman group and the Kamm group to evaluate the efficacy of combinatorial chemotherapies and the transport of chemotherapeutic drugs through vasculature [95,118]. Because chemotherapies are typically delivered intravenously, microfluidic devices demonstrate a more physiologically relevant delivery system. In general, TME models in synthetic hydrogel are suitable platforms for the investigation of chemotherapeutics. Because the TME is known to promote drug resistance, they offer more physiologically relevant data for traditional therapeutics that broadly target multiplying cells. They can also be leveraged to test therapeutics that target specific aspects of the TME that are not present in 2D monoculture while still retaining the advantages of a reductionist, in vitro environment.

## 5. Conclusions

In this review, we discussed many strategies that researchers have used to overcome the limitations of naturally derived hydrogels. Semi-synthetic materials like GelMA and HA-MA combine the biocompatibility of naturally derived hydrogels with greater mechanical tunability. Bioinert synthetic materials like multi-arm PEG and PEGDA allow totally independent biochemical and mechanical modification. We then examined the use of these synthetic materials in models of tumor vasculature and immunology. Current models have helped researchers to investigate the ways in which tumor cells interact with their environment. Some models have also demonstrated potential as physiologically relevant drug screening platforms. Opportunities for future work include the use of microfluidic models for tumor angiogenesis, lymphangiogenesis, and drug screening in synthetic hydrogels. There is also a need to incorporate a broader spectrum of immune cells within static TME models in synthetic hydrogels. Continued advancement in this field will ultimately aid in the understanding of the TME, and will bridge the gap between chemotherapeutic drug efficacies in vitro and in clinical outcomes.
